# Development and feasibility of a mobile experience sampling application for tracking program implementation in youth well-being programs

**DOI:** 10.1186/s13612-016-0038-2

**Published:** 2016-01-21

**Authors:** TanChyuan Chin, Nikki S. Rickard, Dianne A. Vella-Brodrick

**Affiliations:** Melbourne Graduate School of Education, The University of Melbourne, Melbourne, VIC 3010 Australia; School of Psychological Sciences, Monash University, Wellington Road, Clayton, VIC 3800 Australia

## Abstract

Well-being program evaluations mostly focus on identifying effective outcomes rather than measuring the actual extent to which program participants may apply learned skills in subsequent everyday lives. This study examined the feasibility of using a newly developed mobile experience sampling app called Wuzzup to study program implementation in young people participating in well-being programs. Ninety-six participants (60 females; 36 males) between the ages of 13 and 15 years (*M* = 13.87, *SD* = 0.71) were recruited to respond to two random prompts each day, for 7 days, at each of the three data collection time-points. Responses from 69 participants (72 % of initial sample) that met study criteria were retained for analysis. The average response rate was 92.89 %, with an average of 85.92 s to complete each ESM survey. Significant associations between first and second halves of the ESM week, and their respective positive affect and negative affect survey responses, demonstrate internal reliability and construct validity of the Wuzzup app to capture momentary affect and activation states of young people. This study also demonstrated the feasibility and practical utility of the Wuzzup app to profile and track an individual’s learning over time.

## Background

Adolescence is a time of rapid change, development and adjustment from childhood to young adulthood. This transitional phase is filled with new challenges, responsibilities and opportunities for the young person (Aron and Zweig 2003), but at the same time is characterised by a heightened level of behavioural and emotional volatility (Arnett 1999; Hall 1904). During this transition phase, adolescents require support and resources to cope with the changes and develop into healthy, resilient and well-adjusted individuals. Many schools and youth organisations are beginning to implement asset or strengths based programs to not only combat negative mental states but also to bolster well-being (Quinlan et al. 2012). Evaluations of these programs mostly focus on change scores on outcome measures between time-points such as before and after program implementation. However, day to day changes and challenges occur in a myriad of contexts and traditional questionnaire measures do not readily capture the influence of context on a young person’s daily experience. Importantly, rich data on the use (or lack) of strategies to cope with various life events and demands is typically unobtainable with such methods. This means the extent to which young people are applying skills learnt in well-being programs to their daily lives is not being evaluated and therefore program efficacy in terms of practical utility, is difficult to determine.

### Experience sampling methodology

Experience sampling method (ESM) is the repeated sampling of momentary experiences in the individual’s natural environment. Sampling can be of real-time feelings, thoughts and actions in response to the occurrence of everyday events (Brandstatter 1983; Csikszentmihalyi and Larson 1987). ESM provides ecologically valid information on naturally occurring events, experiences and contextual characteristics over time. These details can then be used to build individual profiles to study intra- and inter-personal trends over time.

Compared with retrospective surveys ESM can more accurately capture affect and emotion associated with the studied event. As daily events and experiences vary for individuals, ESM responses collected over time are likely to fluctuate. However, several studies have demonstrated that relative differences of affect ratings between individuals tend to remain fairly stable over time (Csikszentmihalyi and Larson 1987; Eid and Diener 1999; Larson and Csikszentmihalyi 1983). Furthermore, ESM not only minimizes bias associated with recall by collecting in the moment information but also maximizes the validity of the measurement by collecting responses from individuals in their natural environments. ESM is therefore particularly useful in the evaluation of program efficacy and implementation as it provides valuable insight into contextual factors which may facilitate or hinder the application of knowledge obtained from the program being evaluated. Furthermore, ESM is valid for use with young people in a range of contexts, including education settings (Csikszentmihalyi and Larson 1987; Shernoff 2010) and can be delivered effectively through familiar communication tools such as mobile device technology (Reid et al. 2009).

### Mobile experience sampling

The increased availability and utility of portable devices have led to ESM advancements that use electronic data assessment and collection. Mobile technological advancements provide opportunities for researchers to improve real-time sampling across various contexts (Froehlich et al. 2007; Intille 2007). Electronic devices also provide researchers with the added methodological benefit of being able to monitor participants’ response timings through actual date and time stamps. This further strengthens the validity of the collected data.

Mobile devices are increasingly important to young people, with mobile usage rising from 75 % for 12–14 year-olds to 90 % for 15–17 year-olds (Australian Communications and Media Authority 2009). Apart from its popularity, mobile devices also have increasingly powerful capabilities, such as in-built location trackers and accelerometer-based movement sensors, which enable context awareness features (Klasnja and Pratt 2012). This combination of physical proximity to the young person and the technical capabilities of modern mobile devices offer an unprecedented opportunity to obtain in-the-moment feelings and experiences of a young person, making mobile experience sampling an ideal tool for investigating youth participation in mental health and well-being programs.

Despite the methodological and technological advances available for conducting evaluation studies, there is no known existing mobile experience sampling app that has the capacity to track an individual’s uptake and application of skills or knowledge taught in the program of interest. The aim of the current study was to develop a novel mobile experience sampling app utilizing touch screen technology for use with adolescents. We report here the development, feasibility and reliability of using a mobile experience sampling app to track program implementation in a group of youth participants of a youth-led well-being program. By examining completion times, response and completion rates, as well as variation of skills usage over time and across different programs, the feasibility of the app in terms of practical utility can be illustrated. Establishing the reliability and validity of the app for this study via participants’ affect and activation ratings will provide some preliminary psychometric support. Summaries of previous ESM studies conducted with young people indicate that reliability can be assessed by checking consistency of individual ratings over the ESM study week and over time, with correlations in the range of 0.55 and 0.85 indicating a good level of consistency (Csikszentmihalyi 2014; Csikszentmihalyi and Larson 1987). In this study, internal reliability will be examined by correlating affect and activation ESM ratings collected in the first half of the week with the ratings collected over the second half of the week as well as correlating these same ESM ratings collected in the morning with ratings collected in the afternoon. In addition, consistency over time will be examined across three data collection time-points. Validity of ESM measures can be obtained by comparing ESM ratings with independent measures of the same construct and based on Csikszentmihalyi and Larson’s (1987) work correlations in the range of 0.36 and 0.52 would support the construct validity of the mobile experiencing sampling app.

## Method

### Development of a mobile experience sampling app

A new app called ‘Wuzzup’ was developed as part of a comprehensive evaluation of three programs run by the Reach Foundation; a not-for-profit national organization providing community and school-based programs for young people. Their youth-led programs aim to provide young people with a safe space to share their personal experiences and consist of activities that facilitate peer communication and respect. Reach programs are guided by the Positive Youth Development (Lerner et al. 2005) framework, which acknowledges that all young people have strengths and the capacity to be healthy and thriving. Reach also advocates for young people to live authentic lives by being true to their own values and connecting with their feelings. To track program implementation by participants, program facilitators were consulted via focus groups and online surveys to rank and shortlist from a comprehensive list, coping and regulation strategies commonly taught in Reach programs. The top-ranked strategies that were identified by the program leaders were grouped as Reach strategies, and along with other coping strategies identified from the scientific literature, were used as the list of responses to naturally occurring events (pleasant or unpleasant) from which participants could select in the Wuzzup app. The remaining coping strategies were grouped either as “other positive” or “negative” strategies. Grouping was based on previously identified adaptive or maladaptive ways of coping. A point to note here is that the strategies grouped under Reach strategies were not exclusive to Reach per se. However these strategies were explicitly explored in Reach programs, and therefore their level of implementation was examined separately for this study.

### Participants

All participants were recruited through Victorian public secondary schools that expressed interest in running Reach’s youth-led programs. Information about the research and consent forms was provided to relevant school staff, to parents and to students. Young people who provided both assent and parental consent were invited to participate in the evaluation study, which consisted of three data collection time-points (before the program; immediately after the program ended; and between 3 and 6 months after the program ended). Apart from the program participants, students were also recruited from waitlist schools. Students from the waitlist schools contributed as control participants for the entire duration of this study. Control participants were given the option of attending the Reach programs after the research study was completed.

Ninety-six students (60 females; 36 males) between the ages of 13 and 15 years (*M* = 13.87, *SD* = 0.71) initially agreed to participate in the study and all participants (66 program; 30 control) submitted responses during the ESM study week at the first and second data collection time-points. At the third time-point, several students withdrew from the research study (due to a range of personal reasons and withdrawal from school), leaving a final sample of 82 students (85 % of initial sample; 56 program; 26 control).

## Materials

### The *Wuzzup* mobile experience sampling app

Wuzzup, a mobile experience sampling application for use with iOS devices, was developed for the purpose of evaluating program implementation. Wuzzup features a series of questions about current affect and activation states, social and environmental contexts, valence (positive or negative) of a naturally occurring event, responses to events and source of triggers to responses, as well as a subjective evaluation of the response used. Responses to these questions provided detailed information about the individual’s use of strategies to naturally occurring events in day-to-day life and an unprecedented opportunity for the researchers to identify contextual factors which may contribute or hinder the application of skills taught in well-being programs. The app utilizes an intuitive combination of sliding scales, multiple selection options and drop-down lists to collect participant responses efficiently. Participants are also given the option to limit the time range of signal prompts, between their usual waking and bed times, minimizing disruption to the participants’ regular routine.

### Affect and activation

In accordance with the circumplex model of emotion (Russell 1980), both affect and activation were included as measures in this study. Affect was rated on a 7-point Likert-type sliding scale, ranging from 1 (*Unpleasant*) to 7 (*Pleasant*). Activation was also rated on a 7-point Likert-type sliding scale, ranging from 1 (*Passive*) to 7 (*Active*).

### Event valence and type

At each ESM prompt, participants selected the valence and type of event that they had recently experienced (since the last ESM prompt). This information can then be used to ascertain the sort of strategies that young people use to respond to various types of events in their everyday lives. Participants were asked to respond to the following two questions: (1) “What’s the main thing that’s happened to you since you last responded?” with two answer options for event valence (*Something unpleasant happened; Something nice happened*); (2) “What was the event mainly related to?” with six answer options for event type (*Close friend(s); Family; Health and body; Peer(s)/schoolmate(s); School/schoolwork; Work/non*-*school*). The available options were programmed to appear in random order for each ESM prompt to avoid selection bias.

### Strategy use

In response to the event experienced, participants could select from a drop-down list of 18 strategies. An example of strategies explicitly explored in Reach programs is *“I imagined the situation from someone else’s perspective”*. An example of “other positive” strategies is *“I cherished the moment”*. An example of “negative” strategies is *“I tried to avoid the situation”*. The list of strategies (for full list, see Vella-Brodrick et al. 2013) was also programmed to appear in random order for each ESM prompt to avoid selection bias.

### Positive and negative trait affect

Trait affect was measured using the 30-item Positive Affect and Negative Affect Schedule-Child Form (PANAS-C; Laurent et al. 1999). Participants rated the extent to which they generally experience each of the 30 emotions on a 5-point Likert-type scale, ranging from 1 (*Not much or not at* all) to 5 (*A lot*). Published reliability coefficients range between 0.88 and 0.92 (Ebesutani et al. 2011; Laurent et al. 1999). In this study, the average Cronbach’s alpha across three time-points was 0.95 for Positive Affect and 0.94 for Negative Affect.

### Procedure

For each of the three data collection time-points, all participants completed an online PANAS-C survey, and attended a training session conducted by the first author, where research iPods (portable touch-screen electronic devices) were distributed, along with a detailed instruction manual. Research iPods were labeled with each participant’s unique research code and the Wuzzup experience sampling app was uploaded and activated using participants’ research codes. The app was programmed to randomly prompt participants twice each day, once in the morning, and once in the afternoon, for 7 days. After 7 days, research iPods were returned and ESM data were downloaded onto a secure server.

## Results

Data were handled and analysed using IBM SPSS version 21. Data across all three time-points were first screened for non-serious responding, removing responses completed outside what was deemed an acceptable time range of 40–180 s. As per previous ESM studies conducted with adolescents, this study used a 33 % response cut-off rate for each of the three time-points. Based on these criteria, responses from 69 participants (72 % of initial sample; 51 program; 18 control) were retained for analysis.

Participant response and compliance rates were computed to provide an indication of the intrusiveness and burden of participation in this research method. Of the total 14 prompts, participants’ response rate was 96.21 % at Time 1, 94.11 % at Time 2, and 88.36 % at Time 3. Participants took an average of 94.10 s (*SD* = 21.93) to complete each ESM survey at Time 1, 85.71 s (*SD* = 19.62) at Time 2, and 77.96 s (*SD* = 20.83) at Time 3.

### Reliability and validity of affect and activation measures over time

The distribution of the responses was examined using the Kolomogorov-Smirnov test of normality and responses at each of the three time-points were found to be normally distributed. As recommended by Hektner et al. (2007), Z-scores were calculated for ratings of affect and activation to account for variation within individuals. Mean z-scores were computed for each of the following: (1) ratings from the first half of the ESM week; (2) ratings from the second half of the ESM week; (3) ratings in the mornings; (4) ratings in the afternoons. These z-score means for each of the three time-points were then correlated to examine the reliability and validity of the measures over time. Pearson correlations, means and standard deviations of the mean z-scores for each of the three time-points are presented in Table 1.

**Table 1:** Means, standard deviations, and correlations of mean Z-scores

		*M*	*SD*	1	2	3	4	5	6	7	8
Time 1											
1	Affect 1st half	0.27	0.46	–							
2	Affect 2nd half	0.32	0.48	0.45**	–						
3	Affect AM	0.27	0.44	0.82**	0.79**	–					
4	Affect PM	0.32	0.41	0.77**	0.82**	0.77**	–				
5	Activation 1st half	0.23	0.49	0.54**	0.36**	0.48**	0.51**	–			
6	Activation 2nd half	0.29	0.51	0.30*	0.73**	0.49**	0.67**	0.59**	–		
7	Activation AM	0.25	0.51	0.43**	0.60**	0.58**	0.56**	0.85**	0.78**	–	
8	Activation PM	0.27	0.47	0.44**	0.52**	0.41**	0.65**	0.76**	0.85**	0.65**	–
Time 2											
1	Affect 1st half	0.23	0.56	–							
2	Affect 2nd half	0.28	0.57	0.69**	–						
3	Affect AM	0.27	0.51	0.91**	0.87**	–					
4	Affect PM	0.25	0.55	0.87**	0.92**	0.88**	–				
5	Activation 1st half	0.18	0.62	0.69**	0.56**	0.68**	0.64**	–			
6	Activation 2nd half	0.29	0.61	0.58**	0.84**	0.76**	0.75**	0.70**	–		
7	Activation AM	0.23	0.57	0.64**	0.72**	0.75**	0.70**	0.91**	0.89**	–	
8	Activation PM	0.24	0.59	0.71**	0.76**	0.77**	0.78**	0.89**	0.91**	0.90**	–
Time 3											
1	Affect 1st half	0.20	0.52	–							
2	Affect 2nd half	0.20	0.68	0.57**	–						
3	Affect AM	0.20	0.54	0.88**	0.85**	–					
4	Affect PM	0.20	0.56	0.78**	0.93**	0.89**	–				
5	Activation 1st half	0.14	0.66	0.58**	0.33**	0.56**	0.40**	–			
6	Activation 2nd half	0.10	0.73	0.49**	0.79**	0.73**	0.71**	0.70**	–		
7	Activation AM	0.14	0.66	0.57**	0.56**	0.68**	0.55**	0.93**	0.88**	–	
8	Activation PM	0.11	0.65	0.56**	0.65**	0.69**	0.65**	0.86**	0.94**	0.92**	–

** *p* < 0.01; * *p* < 0.05

Both affect and activation ratings from the first half of the ESM week were positively correlated with ratings from the second half of the week and this consistent pattern was observed across all three data collection time-points, demonstrating consistency and reliability of measures over time. Similarly, both affect and activation ratings from morning prompts were strongly correlated with ratings from afternoon prompts at each of the time-points, demonstrating internal reliability of these measures over time.

To establish construct validity, ESM affect ratings were correlated with participants’ PANAS survey responses at each of the three data collection time-points. At all three time-points, participants’ ESM affect ratings from the first and second halves of the ESM week were positively associated with their PA scores (Time 1 first half: *r* = 0.50, *p* < 0.001; Time 1 second half: *r* = 0.58, *p* < 0.001; Time 2 first half: *r* = 0.38, *p* < 0.001; Time 2 second half: *r* = 0.37, *p* < 0.001; Time 3 first half: *r* = 0.31, *p* = 0.01; Time 3 second half: *r* = 0.31, *p* = 0.01) and negatively correlated with their NA scores (Time 1 first half: *r* = −0.28, *p* = 0.02; Time 1 second half: *r* = −0.43, *p* < 0.001; Time 2 first half: *r* = −0.27, *p* = 0.02; Time 2 second half: *r* = −0.28, *p* = 0.02; Time 3 first half: *r* = −0.26, *p* = 0.03; Time 3 second half: *r* = −0.27, *p* = 0.02). These significant correlations between participants’ ESM affect ratings and their PANAS responses provide support for the construct validity of the Wuzzup app to measure affect over time.

### Illustration of variation of skills usage across time

To illustrate the feasibility and utility of the Wuzzup app to track variation of skills usage across different programs over time, selected ESM profiles of program and control participants, across all three data collection time-points, are presented. The aim of using these select profiles is to demonstrate the level of insight that can be gained about how young people respond to life events against the backdrop of learning (or not learning) new skills. This information can be highly useful in understanding the types of events young people identify and their common responses prior to and after well-being interventions so that programs can be refined to meet the real world needs of young people.

**Fig. 1:**
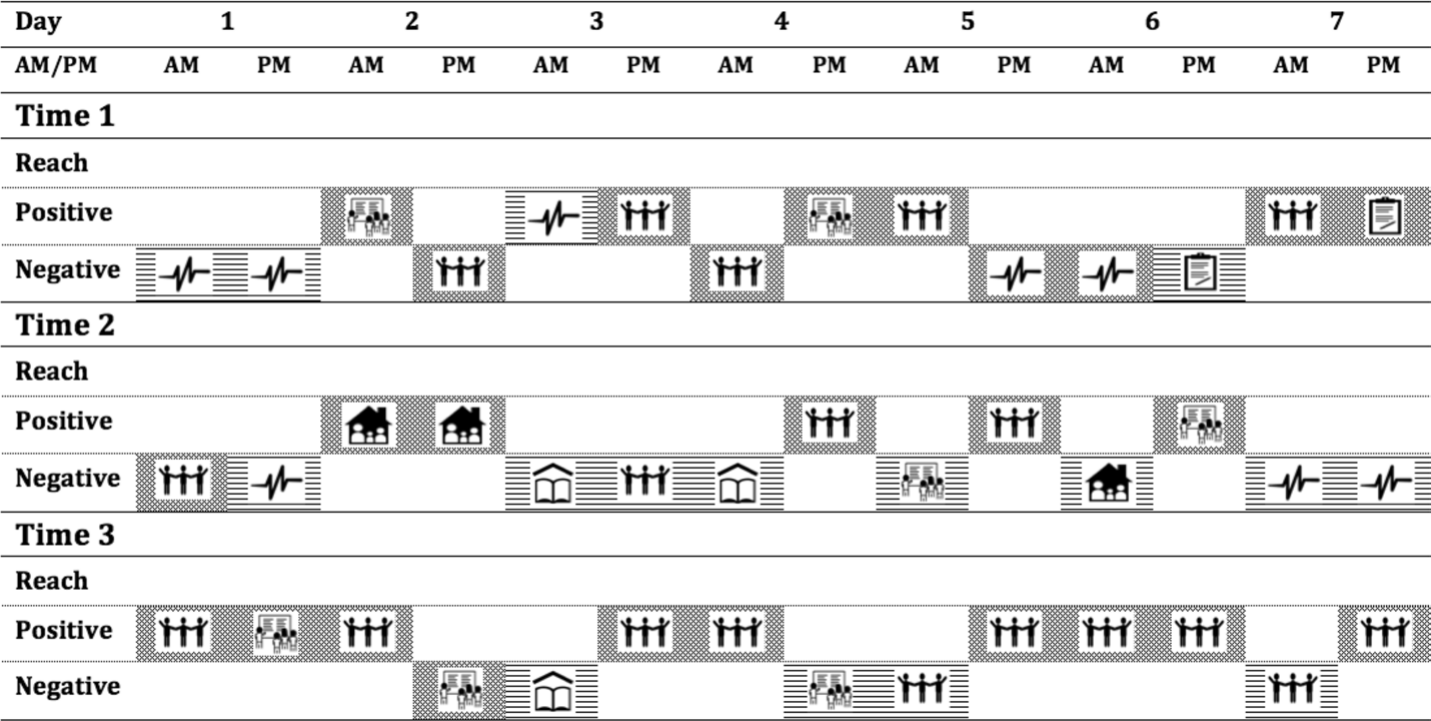
Control participant’s strategy use over time

Each individual profile is a summary of the selected participant’s responses of event valence, event type and strategy use at each of the 14 ESM prompts for all three time-points. Each grid in the profile figure represents the valence and type of event that the participant most recently experienced, and this was then mapped onto the type of strategy they used to respond to the particular event. This ESM profile then provides the ability to track skills usage over time. The profile of a control participant (who did not complete a Reach program) is presented in Fig. 1.

Across the three data collection time-points, this participant used a mix of negative and other positive strategies. It is observed that this participant used mainly positive-type strategies for pleasant events. Negative-type strategies were used for both pleasant and unpleasant events. In relation to the nature of the events, we can see that this young person was reacting negatively to events related to their health and body, as well as school or schoolwork.

**Fig. 2:**
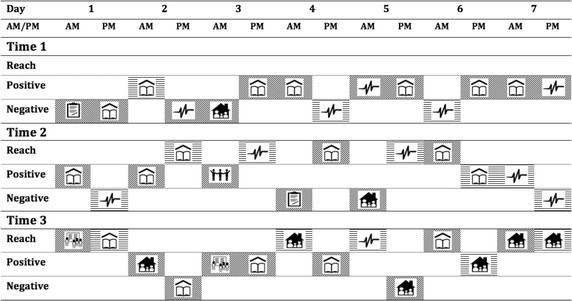
Heroes Day participant’s strategy use over time and in response to reach program participation

The profile of a participant, who attended a Heroes Day Reach program which is a 1-day large-scale workshop that focused on challenging self-perceptions, is presented in Fig. 2.

A general shift of strategy use from negative to mostly Reach and other positive strategies is observed over time in response to participating in a Heroes Day Reach program. In particular, this young person is no longer using negative strategies in response to unpleasant events relating to their health and body.

**Fig. 3:**
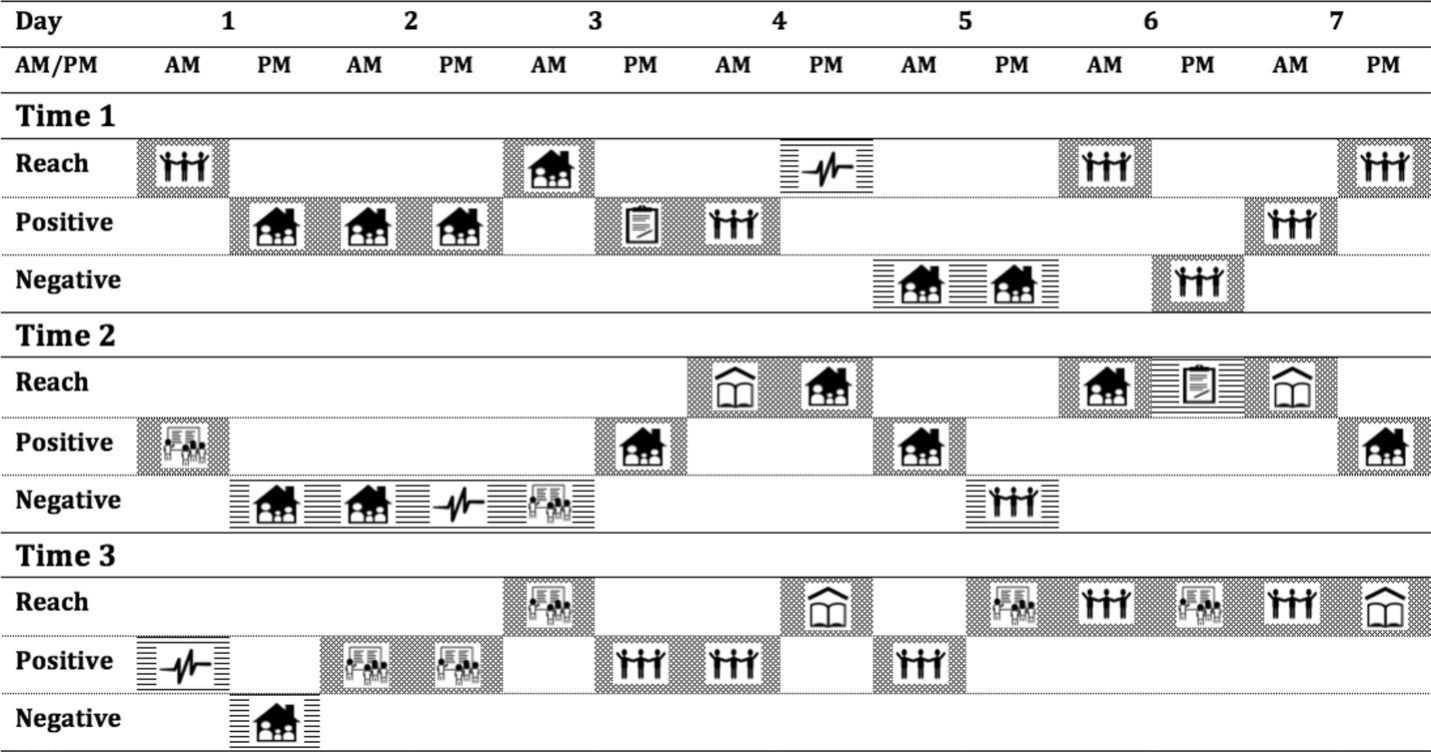
Secondary school workshop participant’s strategy use over time and in response to reach program participation

The profile of another participant, who attended a 90 min Reach school-based workshop that focused on peer relationships in school, is presented in Fig. 3.

This young person indicated the use of all three types of strategies, even before attending the Reach workshop. However, there is a noticeable shift towards use of Reach and other Positive strategies in response to events relating to schoolmates or peers. This is largely reflective of the Reach school workshop, which had the emphasis on improving peer relationships and shifting peer-group dynamics within the school year level. It is a possibility that the Reach school workshop raised awareness for young people to acknowledge and show appreciation towards their peers.

A general pattern across the three ESM profiles was the prevalence of unpleasant events relating to health and body. These individual profiles also reflect the general volatility of the adolescent years and emphasize the need to provide relevant support, across multiple aspects of life, for young people.

## Discussion

This study provides initial evidence that the Wuzzup mobile experience sampling app is a reliable and valid tool for measuring affective and activation states in adolescents. Study findings also demonstrated feasibility of the Wuzzup app to track shifts in strategy use in response to naturally occurring events in everyday life.

Previous ESM studies examining the reliability and validity of average mood responses between the first half and second half of the ESM week have reported correlation coefficients of between 0.55 and 0.85 (Csikszentmihalyi 2014). In this study, average affect responses between the first half and second half of the ESM week were also positively correlated with a similar, albeit slightly lower coefficient range (0.45–0.69). In addition, this study measured activation, and responses from the first half and second half of the ESM week were also positively correlated, with coefficients of between 0.59 and 0.70. As an extension to previous ESM methodology, this study also examined the affect and activation ratings from the morning and afternoon prompts. Participants’ affect and activation ratings from these two time prompts were also strongly correlated, with coefficients of between 0.77 and 0.89 for affect, and 0.65 and 0.92 for activation. In addition, across all three time-points, participants’ ESM affect ratings from the first and second halves of the ESM week were positively correlated with their PA scores and negatively associated with their NA scores. These significant associations, across time, demonstrate construct validity and internal reliability of the Wuzzup app to capture momentary affect and activation states of young people.

The individual ESM profiles not only provide valuable information about the nature of life events that young people face in daily lives, but also highlight the need to use reliable measures that capture various aspects of life experiences. This study also demonstrated the usefulness of the Wuzzup app in tracking program participants’ use of strategies, identifying key areas in which participants were able (or not) to apply taught strategies and other areas requiring further training or support. The individual ESM profiles illustrate that the Wuzzup app is a sensitive measure of behaviour change and it can provide rich within-participant data, making it an ideal tool for evaluating well-being program implementations.

As with most ESM studies, there were several challenges with the development and use of the Wuzzup app. As the Wuzzup app was not publicly available on the App Store, a significant proportion of time was required to prepare the research iPods for data collection. Even though participants took an average of 86 s to complete each ESM survey, they were committed to respond to 14 ESM surveys at each of the three, week long data collection time-points. To minimize participant attrition, the research team maintained close contact with participants throughout the study, and conducted prize draws for $20 iTunes gift cards at each data collection time-point. Sample representativeness could be a concern for mobile experience sampling studies as ownership of suitable mobile devices would be a significant limiting factor. Participation in this study was not restricted to individuals having appropriate devices as research iPod touch devices were loaned to student participants for the duration of the ESM study week. However, this presented different issues for the research team as some students did misplace or drop their research iPod, resulting in the immediate need to replace the test unit. It is therefore essential to have several spare research iPods on standby in participating research schools for immediate replacement. Moving forward, there is also opportunity to assess and compare research uptake and response rates when participants have the option of either using experimenter-provided research iPods or installing the Wuzzup app on their personal mobile devices.

There are many benefits of using the Wuzzup app to track program implementation in well-being programs. Firstly, it strengthens the multi-method approach in an evaluation study by supplementing traditional self-report survey responses and focus group interviews. Secondly, it is a sensitive measure of fluctuation and change, and can therefore be used to effectively profile an individual’s learning or progress, for monitoring change within and between program participants. Thirdly, the use of intuitive and quick-response type features of the Wuzzup app makes it relatively unobtrusive for use in daily life contexts for young people in school settings. Lastly, the Wuzzup app is highly versatile and can be tailored for different programs. These benefits clearly outweigh the challenges of using the Wuzzup mobile experience sampling app.

In summary, the Wuzzup app has been shown to be a reliable and valid tool for measuring affect and activation in young people. This study has also shown that the Wuzzup app is an effective tool for profiling and tracking an individual’s learning over time. The Wuzzup app is a valuable contribution to the measurement and understanding of everyday life experiences of young people. Importantly, it has enhanced the capability to track usage of skills taught in well-being programs and this will allow future research to identify and generate a better understanding of the key components of effective well-being programs for young people.
